# Alterations in the mucosa-associated fungal microbiota in patients with ulcerative colitis

**DOI:** 10.18632/oncotarget.22534

**Published:** 2017-11-20

**Authors:** Xinyun Qiu, Jingjing Ma, Chunhua Jiao, Xiaqiong Mao, Xiaojing Zhao, Meijiao Lu, Kai Wang, Hongjie Zhang

**Affiliations:** ^1^ Department of Gastroenterology, First Affliated Hospital of Nanjing Medical University, Nanjing, Jiangsu 210029, China; ^2^ Institute of Apicultural Research, Chinese Academy of Agricultural Sciences, Beijing 100093, China

**Keywords:** ulcerative colitis, intestinal fungi, mucosal-associated microbiota, mucosal inflammation, high-throughput sequencing

## Abstract

**Background:**

Fungi colonize the human gut and might play a key role in the pathogenesis of ulcerative colitis (UC). However, studies on the fungal composition in the gut (especially adhering to the intestinal mucosa) of UC patients is limited.

**Results:**

The number of fungi decreased significantly in inflamed mucosa compared with that in HS mucosa. Fifteen major genera were examined, among which *Wickerhamomyces*, unidentified genus of *Saccharomycetales*, *Aspergillus*, *Sterigmatomyces*, and *Candida* showed increasing trends, whereas *Exophiala*, *Alternaria*, *Emericella*, *Epicoccum*, *Acremonium*, *Trametes*, and *Penicillium* showed decreasing trends in UC patients compared to the HS. The pro-inflammatory cytokines (IL-Iβ, TNF-α, INF-γ, IL-6, IL-17A, and IL-23) were up-regulated in the UC group. The genera *Wickerhamomyces*, *Nigrospora*, and *Penicillium* were positively correlated, while *Sporobolomyces* and *Trametes* were negatively correlated with the expression of several colonic pro-inflammatory cytokines and the Baron and/or Mayo score.

**Conclusions:**

Our study confirms the alteration of the colonic fungal microbiota in the UC patients, which might be associated with mucosal inflammation and pathogenesis of UC. Further studies need to identify the roles of different intestinal fungi in detail, and to determine the mechanism of the host-fungal interaction underlying the development of UC.

**Methods:**

Mucosal samples of inflamed descending colon from 14 active UC patients and 15 healthy subjects (HS) were analyzed by high-throughput sequencing to compare the fungal microbiota. The expressions of pro-inflammatory cytokines (IL-Iβ, TNF-α, INF-γ, IL-6, IL-17A, and IL-23) in intestinal mucosal tissues were examined. The Baron and Mayo scores of UC patients were evaluated, and the correlation between intestinal fungal composition and intestinal inflammatory status was analyzed.

## INTRODUCTION

Ulcerative colitis (UC), a major type of inflammatory bowel disease (IBD) [1], is a chronic inflammatory disorder that is limited to the colon and rectum. UC is characterized by alternating stages of clinical activity and remission [2], and its exact pathogenesis remains unclear. However, previous studies have demonstrated that UC involves an aberrant immune response to the gut microbiota in genetically susceptible hosts [3], and environmental factors also participate in the development of gut inflammation [4]. The bacterial microbes involved in IBD have been widely studied. Increased abundance of bacteria belonging to the *Bacteroidetes* and *Proteobacteria* phyla and decreased abundance of bacteria belonging to the *Firmicutes* phylum have been observed in IBD patients [5]. Growing evidence have suggested the hypothesis that intestinal dysbiosis result in the immune impairment associated with IBD [5, 6]. In addition to bacteria, gut microbiota including fungi, viruses, bacteriophages, and archaea also colonize the gastrointestinal tract and some of these communities are also markedly shifted in IBD [7], although their compositions have not been thoroughly examined.

The fungi are a group of eukaryotic organisms that colonize the gut of mammals [6]. Although the number of fungi in the gut is much lower than that of bacteria, the volume of a typical fungal cell is much larger than that of a typical bacterium (approximately 100-fold larger) [8]. Further, fungi can provide many unique metabolic materials to bacteria [8]. Several studies have reported close relationships between intestinal fungi and human intestinal bowel disease. An antibody against fungi (anti-*Saccharomyces cerevisiae* antibody, ASCA) is considered to be a discriminating biomarker for Crohn's disease (CD) [9]. Moreover, the severe form of UC is strongly associated with a polymorphism of the Dectin-1 gene, which encode a receptor that recognizes the β-1,3-linked and β-1,6-linked glucans from fungi [10]. Mice lacking Dectin-1 have increased susceptibility to chemically induced colitis because of the altered responses to indigenous intestinal fungi [11]. Our previous studies have shown that fungus-depleted mice are more susceptible to DSS-induced colitis [6, 12], demonstrating the important role that fungi play in the mammalian gut.

The gastrointestinal microbiota can be divided into two ecosystems [13]: the luminal microbiota in the feces and the mucosal microbiota present in the intestinal epithelium. Because of its close proximity, the mucosal microbiota is more likely to be involved in intestinal diseases than its luminal counterpart [12]. However, owing to the ease of acquisition and ethical considerations, most previous studies examined the fecal microbiota rather than the mucosal-associated counterpart. Sokol *et al.* [14]. reported an increased Basidiomycota/Ascomycota ratio, an increased proportion of *Candida albicans*, and a decreased proportion of *Saccharomyces cerevisiae* in fecal samples from IBD patients compared with samples from the HS. Li *et al.* [15]. analyzed the ileal mucosal and fecal fungal compositions of patients with Crohn's disease and found higher proportions of *C. albicans*, *Aspergillus clavatus*, and *C. neoformans* compared with those in HS. Lewis *et al.* [16]. examined pediatric CD subjects and reported a positive association between five fungal taxa (*Saccharomyces cerevisiae*, *Clavispora lusitaniae*, *Cyberlindnera jadinii*, *C. albicans*, and *Kluyveromyces marxianus*) and CD development. Currently, studies based on colonic mucosal samples from UC patients are limited. Our previous studies showed that the fecal fungal microbiota is significantly different from its mucosal counterpart [12].

In this study, in order to investigate whether there is dysbiosis of the fungal microbiota in UC patients, we analyzed the fungal microbiota of the colonic mucosa in both UC patients and HS using high-throughput sequencing technology. In addition, the association between intestinal fungi and intestinal inflammation was assessed to uncover the link between the fungal microbiota and IBD pathogenesis.

## RESULTS

### Characteristics of the study participants

In this study, we collected mucosal samples from 14 UC patients (6 females and 8 males) and 15 normal healthy controls (7 females and 8 males). The mean age of UC patients was 40.36 ± 13.27 and for of healthy controls was 42.40 ± 14.65. Left-sided, procto-sigmoid, and extensive colitis were present in 6, 2, and 6 patients, respectively. The Baron and Mayo scores and the concomitant medications for UC patients are summarized in Table 1. The detailed clinical characteristics of the UC patients and controls are summarized in Table 2.

**Table 1:** Scoring criteria of Mayo and Baron score

Mayo score	Baron score
**Stool frequency** 0 = Normal number of stools 1 = 1–2 stools more than normal; 2 = 3–4 stools more than normal; 3 = 5 or more stools more than normal.	0 = denoting normal mucosa; 1 = granular mucosa with an abnormal vascular pattern; 2 = friable mucosa; 3 = microulceration with spontaneous bleeding; 4 = gross ulceration.
**Rectal bleeding** 0 = No blood seen; 1 = Streaks of blood with stool less than half the time; 2 = Obvious blood with stool most of the time; 3 = Blood alone passed	
**Findings of flexible proctosigmoidoscopy** 0 = Normal or inactive disease; 1 = Mild disease (erythema, decreased vascular pattern, mild friability); 2 = Moderate disease (marked erythema, absent vascular pattern, friability, erosions); 3 = Severe disease (spontaneous bleeding, ulceration)	
**Physician's global assessment** 0 = Normal; 1 = Mild disease; 2 = Moderate disease; 3 = Severe disease.	

**Table 2:** Characteristics of the study participants

	UC (*n* = 14)	Normal (*n* = 15)
Sex (female/male)	6/8	7/8
Age (year ± SD)	40.36 ± 13.27	42.40 ± 14.65
Disease sites	Left-sided: *n* = 6	
	Proctosigmoid: *n* = 2	
	Extensive : *n* = 6	
Baron score	0:*n* = 0	
	1:*n* = 5	
	2:*n* = 3	
	3:*n* = 3	
	4:*n* = 3	
Mayo score	2:*n* = 3	
	3:*n* = 3	
	4:*n* = 2	
	6:*n* = 1	
	10:*n* = 3	
	11:*n* = 2	
Concomitant medications	Mesalazine:*n* = 10	
	None:*n* = 4	
	AZA/6-MP:*n* = 0	
	Probiotics and/or antibiotics and/or anti-fungal agent use (within 8 weeks) *n* = 0	

### Quantification and diversity of the fungal microbiota in UC patients and controls

**Figure 1:**
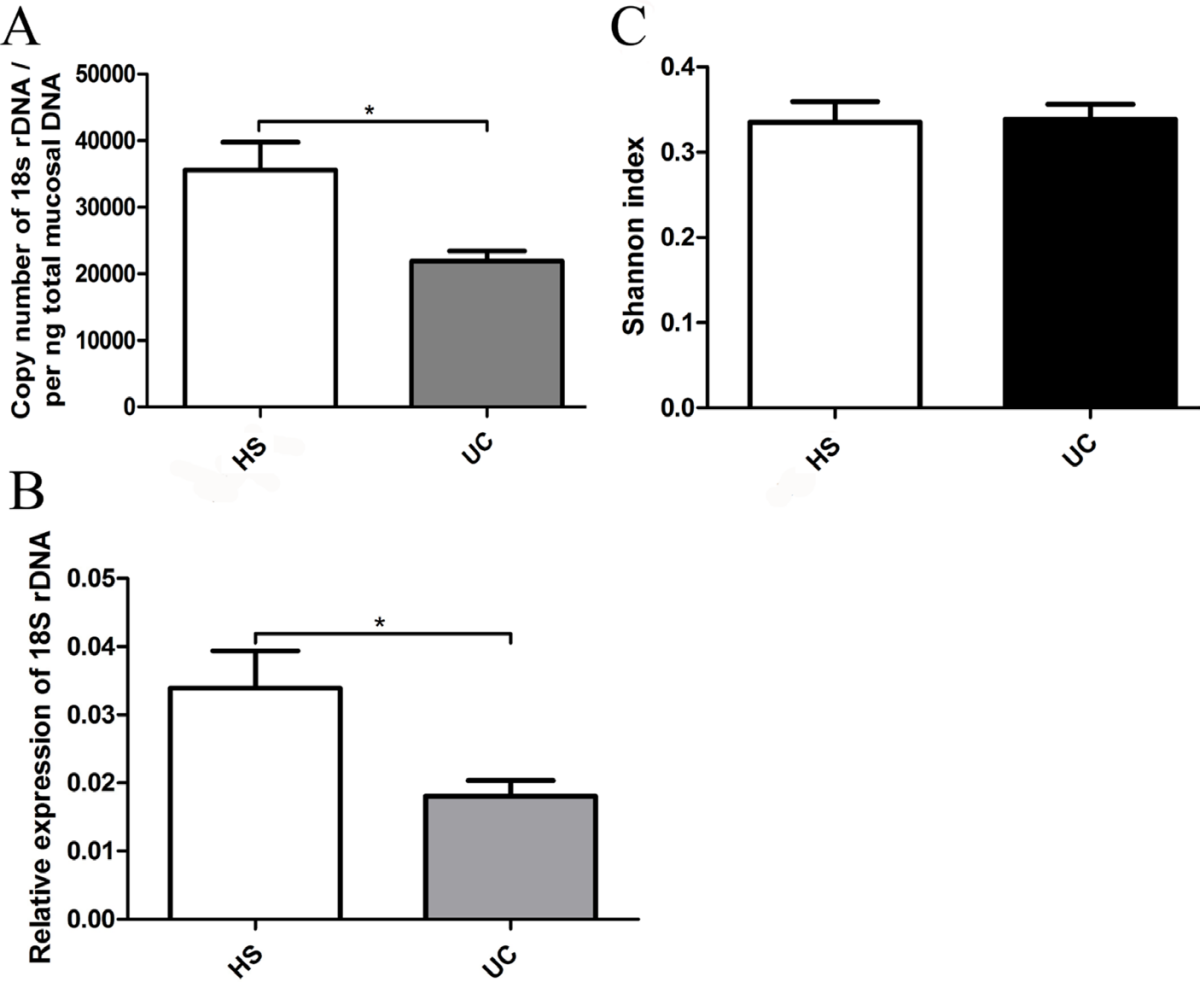
(**A**) qRT-PCR of 18S rDNA was performed on per ng total DNA isolated from the mucosal specimens of HS and UC patients. (**B**) Relative expression of 18S rDNA level in mucosal specimens of HS and UC patients measured by qRT-PCR and normalized to RPLPO. (**C**) The Shannon-Weiner biodiversity index (Shannon index) was measured to represent the diversity of fungi in the gut.

We used 18S rDNA primer-amplified fragments to quantitatively analyze the fungal contents in the gut. The 18S rDNA (fungal) content was found to be significantly lower in UC patients compared with that in the HS by absolute and relative quantitative PCR (Figure 1A and 1B). However, the fungal Shannon biodiversity index values were similar between the two groups (Figure 1C).

### Taxonomy of the intestinal mucosal-associated fungal microbiota in UC patients and HS

We analyzed the gut fungal microbiota in the mucosal samples from UC patients and HS based on ITS1-2 rDNA sequencing. After application of strict trimming criteria to exclude low-quality reads, a total of 13,150,816 reads remained, with an average of 45,373 reads per sample. The rarefaction curve that was used to visualize the observed number of OTUs on the sequence counts was saturated, indicating that no new OTUs would be found even if the sequencing depth was increased (Supplementary Figure 1).

### OTU-level analysis of the overall fungal microbiota composition in the colonic samples from UC patients and HS

**Figure 2:**
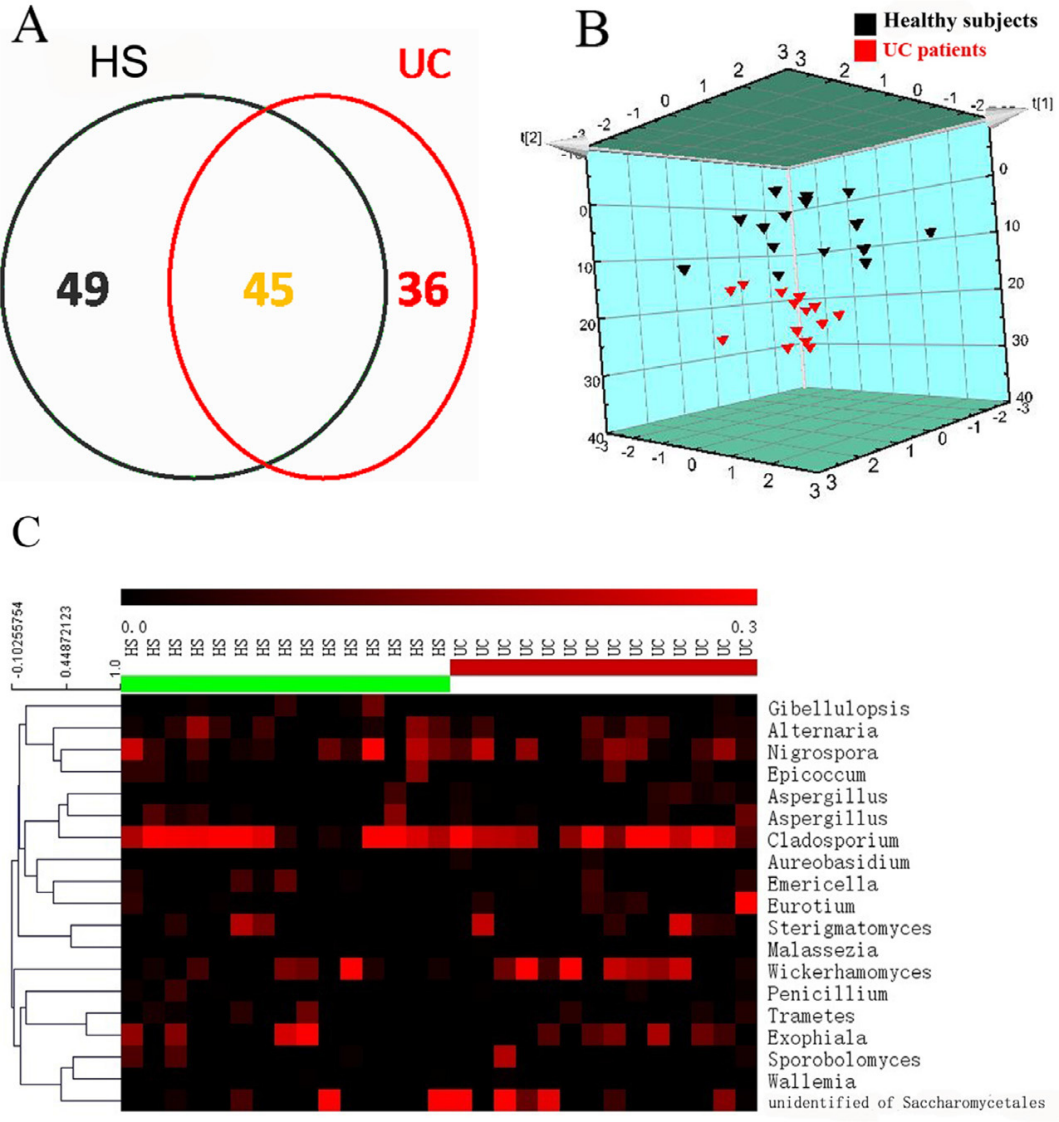
Fungal compositions vary in colonic mucosa between healthy subjects (HS) and UC patients (**A**) Fungal operational taxonomic units (OTUs) (97% similarity level) number in colonic mucosa of HS and UC patients. (**B**) Partial least-squares discriminant analysis (PLS-DA) scores plot based on the relative abundance of fungal OTUs in colonic mucosa of HS and UC patients. (**C**) Heat maps showing the 19 core OTUs of fungal communities inferred from colonic mucosa, with each subject shown individually. Each vertical lane corresponds to a subject, and the colored squares in each row indicate the relative abundance of the core OTU among the 29 subjects.

We defined 130 OTUs in the descending colonic samples after the OTUs with less than 10 supported reads were removed, according to a method described previously [17]. In total, 45 OTUs overlapped between the HS and UC patients, and 49 and 36 OTUs were uniquely present in the HS and UC patients, respectively (Figure 2A). PLS-DA score plots (Figure 2B) and heatmaps (Figure 2C) based on the core OTUs (Supplementary Table 1) revealed that the colonic mucosal fungal community in the HS and UC patients could be classified into two different clusters, which suggested that fungal compositions in UC patients were different from that in HS.

### Shifts in the fungal communities in inflamed intestinal mucosa

**Figure 3:**
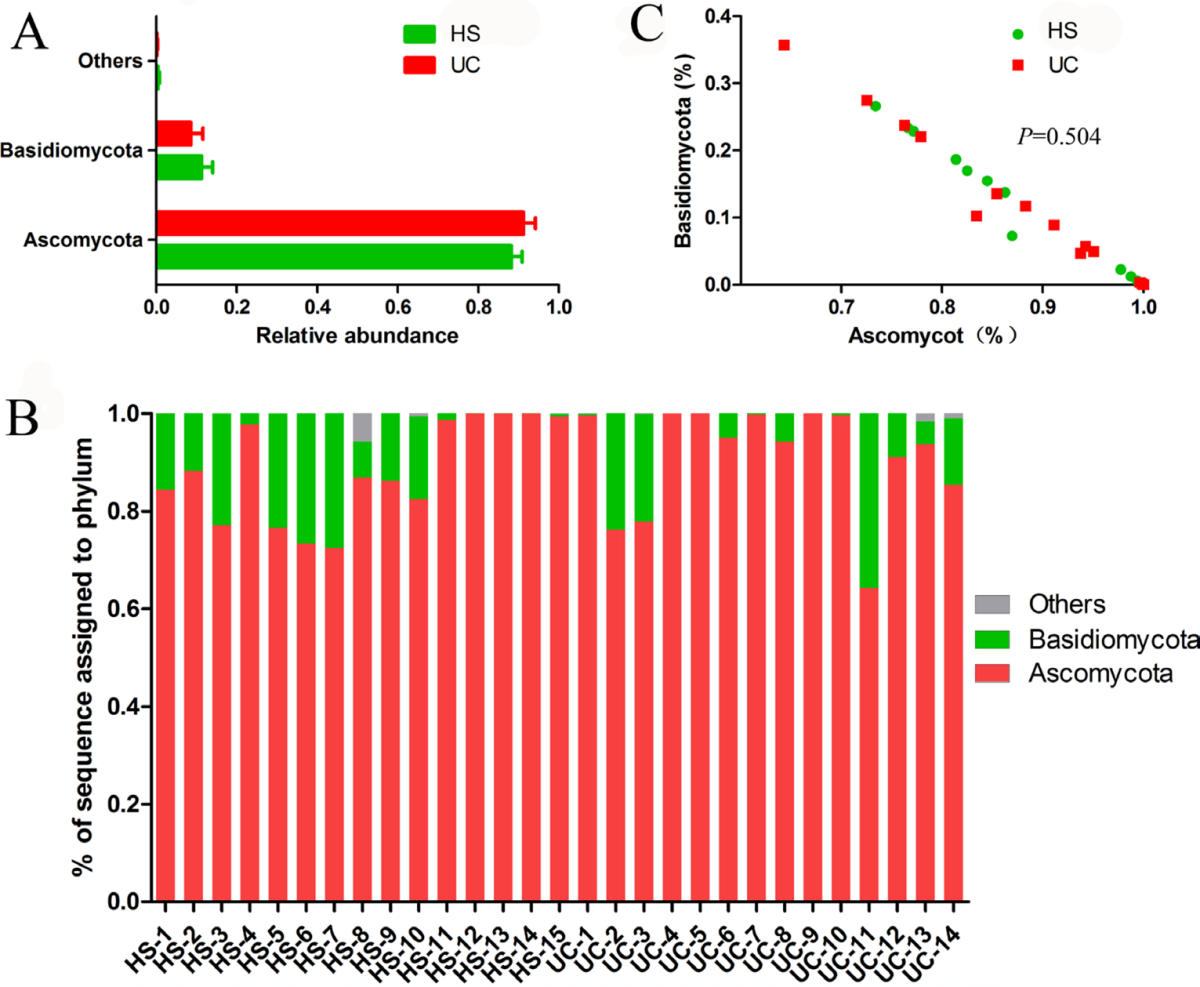
Distribution of fungi in colonic mucosal samples at the phylum level, exhibited integrally (**A**) and individually (**B**). And the Basidiomycota/Ascomycota relative abundance ratio in HS and UC groups were studied (**C**).

**Figure 4:**
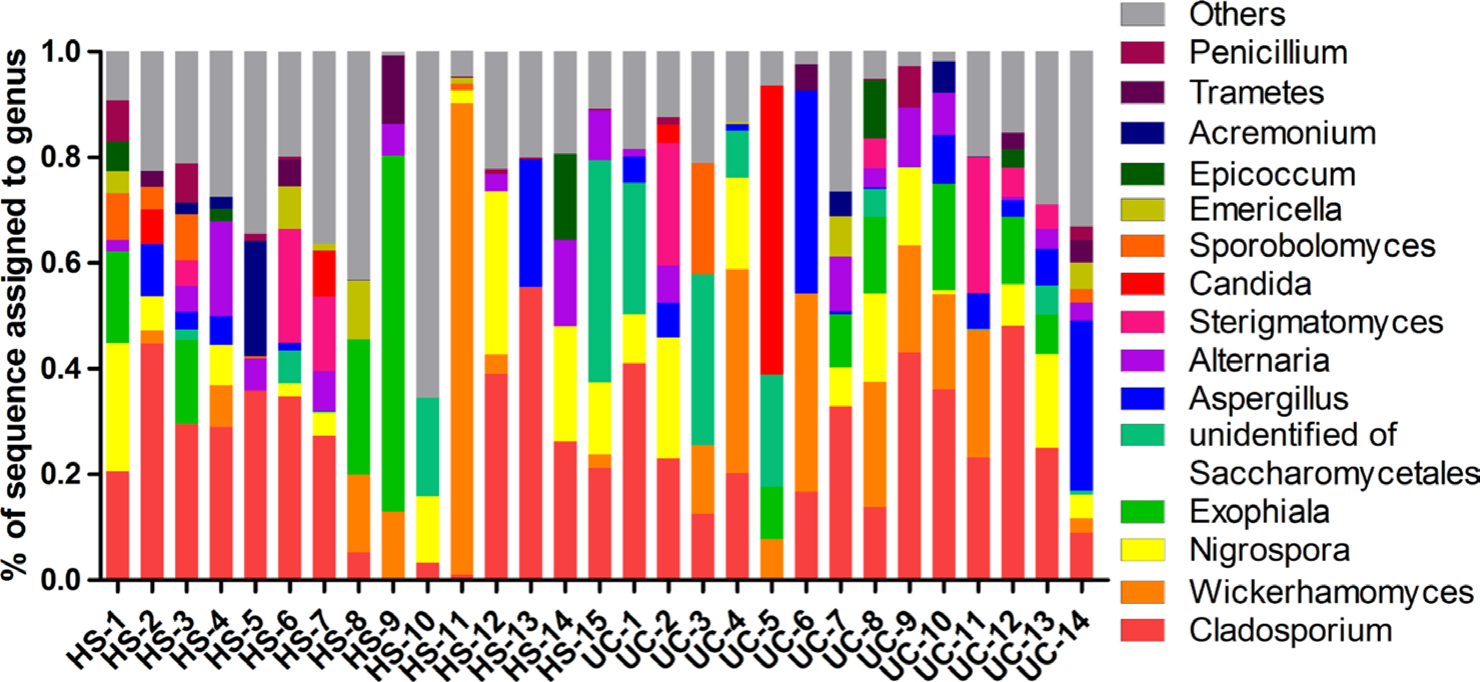
Distribution of fungi in colonic mucosal samples of HS and UC patients at the genus level

**Figure 5:**
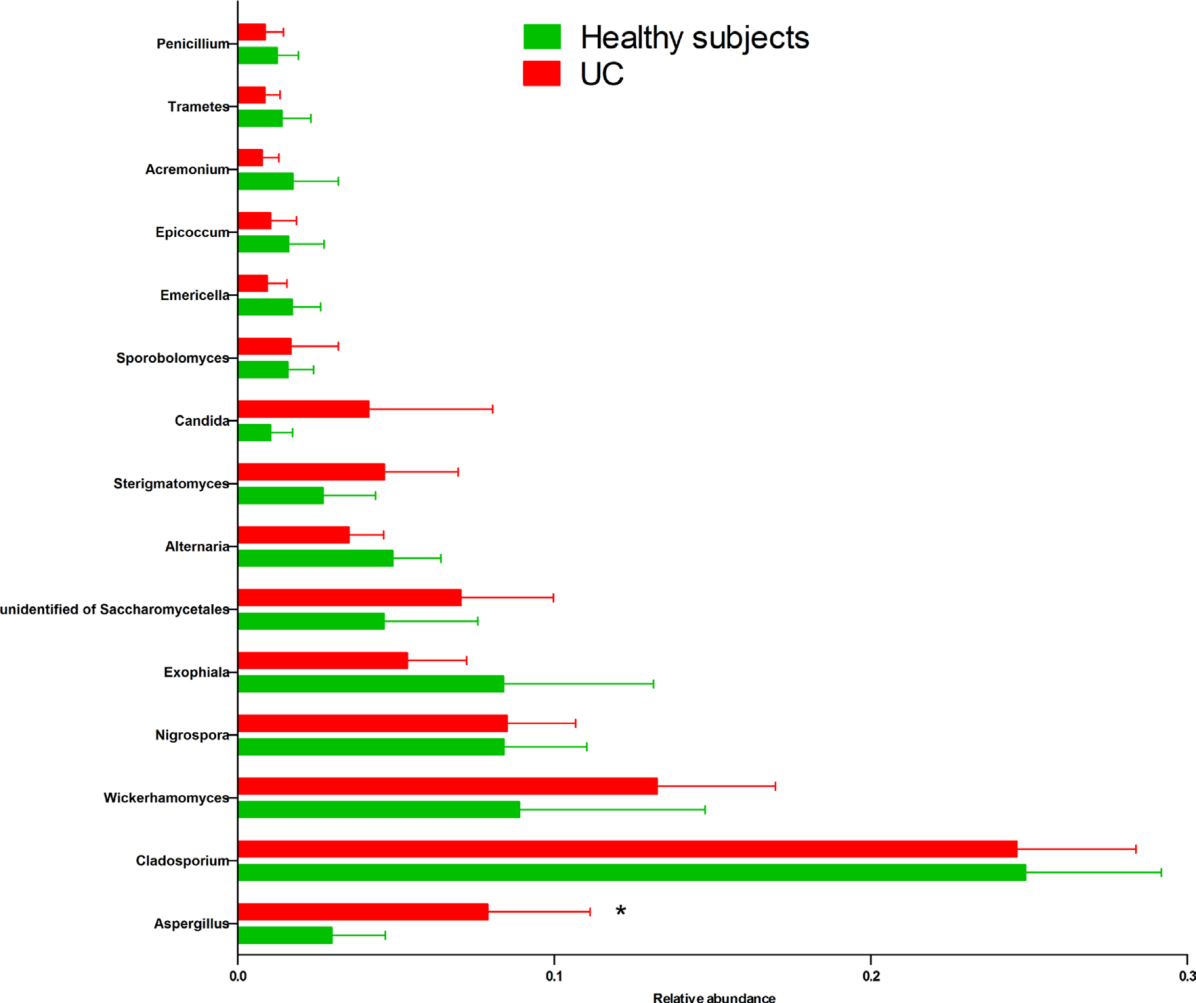
Comparison of 15 main fungal genera (relative abundance ≥ 0.01 on average) in the colonic mucosa of HS and UC patients ^*^P < 0.05.

Two dominant phyla (*Ascomycota* and *Basidiomycota*) accounted for more than 99% of the population in all of the samples (Figure 3A–3B). The proportions of *Ascomycota* and *Basidiomycota* were not significantly different between the two groups (Figure 3C). In addition, 15 major genera (*Cladosporium*, *Wickerhamomyces*, *Nigrospora*, *Exophiala, Aspergillus*, *Alternaria*, *Sterigmatomyces*, *Candida*, *Sporobolomyces, Emericella*, *Epicoccum*, *Acremonium*, *Trametes*, *Penicillium*, and unidentified genus of *Saccharomycetales*) were detected in the colonic mucosa of the HS and UC patients (Figures 4 and 5). *Wickerhamomyces*, an unidentified genus of *Saccharomycetales, Aspergillus*, *Sterigmatomyces*, and *Candida* showed an increasing trend in UC patients compared with that in the HS; among these fungi, there was a marked difference in *Aspergillus* between the groups. In contrast, *Exophiala, Alternaria*, *Emericella*, *Epicoccum*, *Acremonium*, *Trametes*, and *Penicillium* showed decreasing trends in UC patients without significant differences compared to the HS. In addition, the proportions of *Cladosporium*, *Nigrospora*, and *Sporobolomyces* were similar between the two groups (Figures 4 and 5).

### Correlation between intestinal fungal composition and mucosal inflammatory status

**Figure 6:**
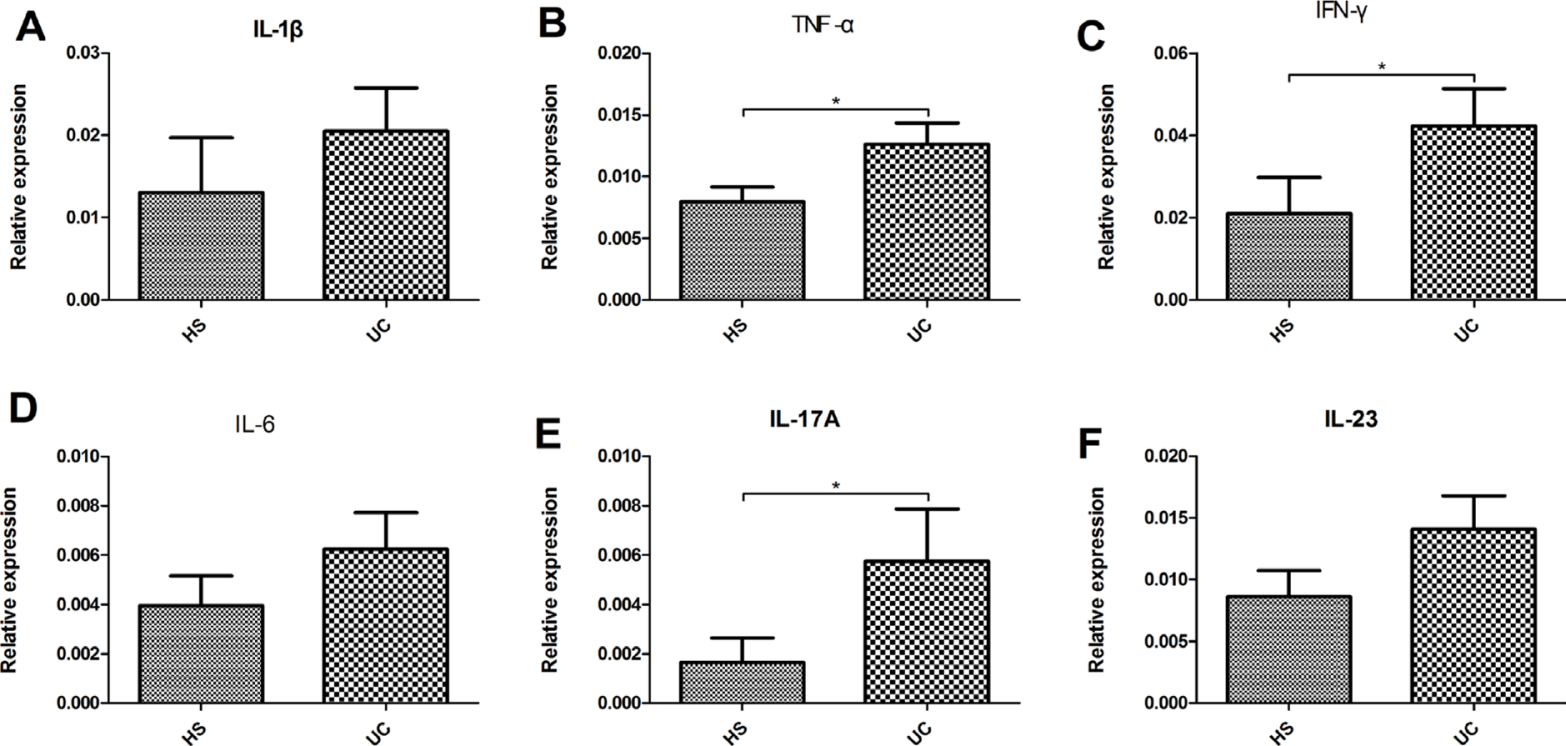
Relative expression of pro-inflammatory cytokine (IL-1β (**A**), TNF-α (**B**), IFN-γ (**C**), IL-6 (**D**), IL-17A (**E**), and IL-23 (**F**)) levels in the colonic mucosa of HS and UC patients. ^*^P < 0.05.

**Figure 7:**
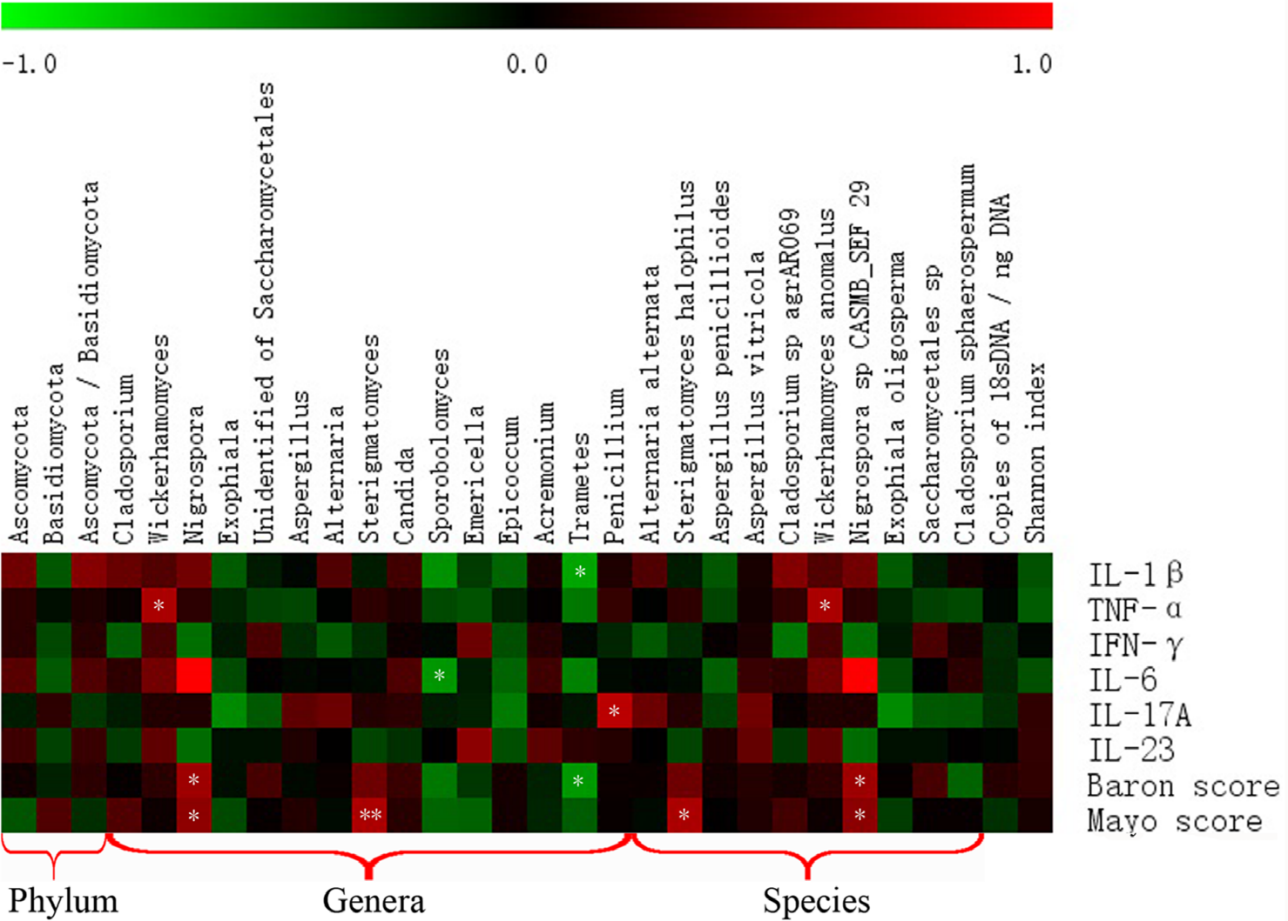
Correlation between intestinal fungal composition and intestinal inflammatory status Red box indicate positive correlation, green box indicate negative correlation. The darker color indicates stronger correlation. ^*^P < 0.05, ^**^P < 0.01.

We evaluated the intestinal fungal composition at the phylum and genus levels and analyzed its correlations with several mucosal inflammatory cytokines (IL-Iβ, TNF-α, INF-γ, IL-6, IL-17A, and IL-23) and Baron and Mayo scores in UC patients. The expression of all six cytokines showed an increased trend in the gut of UC patients compared with that in the HS, and the expressions of TNF-α, INF-γ, and IL-17A between two groups showed significant differences (Figure 6). In addition, we analyzed the association between the intestinal fungal composition and the expressions of inflammatory cytokines, Baron score, and Mayo score, respectively. The proporation of *Ascomycota, Basidiomycota* and the ratio of *Ascomycota* to *Basidiomycota* did not show significant correlation with the intestinal inflammatory level. The genus *Wickerhamomyces* (the major species was *Wickerhamomyces anomalus*) and *Penicillium* were positively correlated with expression of TNF-α (*r* = 0.667, *P* = 0.018) and IL-I7A (*r* = 0.734, *P* = 0.007), respectively. The *Nigrospora* (the major species was *Nigrospora sp CASMB_SEF 29*) showed a positive correlation with the clinical Baron index (*r* = 0.627, *P* = 0.016) and Mayo score (*r* = 0.561, *P* = 0.037) and the *Sterigmatomyces* (the major species was *Sterigmatomyces halophilus*) also showed a positive correlation with the Mayo score (*r* = 0.734 *P* = 0.007). Interestingly, *Sporobolomyces* was negatively correlated with the expression of IL-6 (*r* = –0.591, *P* = 0.043) and *Trametes* was negatively correlated with the IL-1β (*r* = −0.650, *P* = 0.022) and Baron index (*r* = −0.599, *P* = 0.023) (Figure 7).

## DISCUSSION

The involvement of the fungal microbiota in the etiology of UC remains unclear. Because of the difficulty in obtaining specimens, most previous studies have evaluated the intestinal microbiota using fecal samples, rather than samples from the intestinal mucosa. In this study, we determined the characteristics of the intestinal fungal communities adhering to the colonic mucosa of HS and UC patients and found different micro-ecosystems between the two groups. The global fungus load was significantly decreased in the gut of UC patients compared to the normal controls (Figure 1A and 1B), which was quite different from the result reported in CD patients [18]. Interestingly, we did not observe any significant difference in Shannon index between the two groups (Figure 1B). After further exploration of the global structure of the fungal microbiota at the OTU level, 94 OTUs were identified in the HS, whereas only 81 were found in UC patients, reveling a tendency for lower diversity of the fungal microbiota in the inflammatory gut compared with that in the normal gut [16]. The PLS-DA (Figure 2B) and heat-map (Figure 2C) based on the 19 core OTUs (OTUs present in more than 33.3% of the UC and/or HS mucosal samples) revealed a marked separation of fungal composition between the HS and UC patients. The heat-map indicate that one core OTU assigned to the genera *Wickerhamomyces* was more widely present in the gut of UC patients compared with the HS. However, as the sample size in the study was not large, complete data for the human intestinal microbial could not be obtained in this study. Further, additional work is needed to elucidate the difference between patients with UC and HS more comprehensively.

We next studied the fungal communities at the phylum and genus levels. No significant differences in the two predominant phyla (Ascomycota and Basidiomycota) were observed between the HS and UC patients (Figure 3A and 3B). Moreover, both phyla were strongly and negatively correlated with each other in the gut of subjects (Figure 3C). The abundance of Ascomycota and Basidiomycota and the ratio of Ascomycota to Basidiomycota did not differ between the HS and UC patients, indicating that the differences in the fungal composition between the two groups were not occurring at the phylum level in mucosal specimens. This is different from the result reported by Sokol *et al*. [14], who found an increased Basidiomycota/Ascomycota ratio in fecal samples of IBD patients compared with that in the HS. This discrepancy might be due to the different distribution of fungal microbiota between mucosa and feces.

We further studied the fungi at the genus level. Fifteen major genera (abundance ≥ 0.01) were found, among which *Aspergillus* was significantly increased in the UC patients compared with that in the HS (Figure 4). *Aspergillus* is an ubiquitous fungus that is abundant throughout the gastrointestinal tract and has been shown to produce the carcinogenic mycotoxins aflatoxin, and ochratoxin [19]. Numerous species of *Aspergillus* have been reported in the gastro-intestinal tract of humans, these are known to cause opportunistic infections in immunocompromised UC patients [20, 21]. Moreover, invasive gastrointestinal *Aspergillosis* has been reported to cause abdominal pain and gastrointestinal bleeding [20]. Thus, we consider that the increase in *Aspergillus* could play an important role in gut inflammation in UC patients. In addition, although the proportion of *Candida albicans* was reported to be higher in fecal samples of UC patients compared with that in healthy controls in previous studies [14], this genus was not significantly over-represented in the mucosal samples (but exhibited an increased trend) in this study (Figure 5). The lack of significance may be attributed to the limitations arising from the small sample size. Finally, the fungal composition did not show significant differences between the groups at the species level (Supplementary Figure 2).

Previous studies have documented that the intestinal fungi can be recognized by the membrane-bound receptors (e.g., lectin receptors, Toll-like receptors, scavenger receptor family members etc.) of many immunocytes [22]. These receptors further trigger the phagocytosis, respiratory burst, and intracellular signaling pathways, causing the release of multiple pro-inflammatory cytokines following activation by the intestinal fungi [22, 23]. Several pro-inflammatory cytokines (IL-Iβ, TNF-α, INF-γ, IL-6, IL-17A, and IL-23) [24] are up-regulated in the gut of UC patients and were considered to be significantly associated with the severity of UC (Figure 6). Thus, we further investigated the association between intestinal fungi and mucosal pro-inflammatory cytokines, as well as the clinical Baron and Mayo scores.

The fungal composition is not significantly correlated with the intestinal inflammation at the phylum level. When the fungi are studied at the genus and species levels, we found that the genera *Wickerhamomyces* (mainly *Wickerhamomyces anomalus*) *and Penicillium* were positively associated with the expression of TNF-α and IL-17A, respectively (Figure 7). Two genera (Nigrospora [mainly Nigrospora sp CASMB_SEF 29] and Sterigmatomyces) were positively associated with the clinical Baron index and/or Mayo score (Figure 7). However, *Sporobolomyces was* negatively correlated with the expression of IL-6 and the *Trametes* was negatively correlated with the intestinal IL-1β level and clinical Baron score (Figure 7).

To the best of our knowledge, no previous study has reported the relationship between *Wickerhamomyces*, *Sterigmatomyces*, and *Nigrospora* and intestinal inflammation. Further, the mechanisms by which fungi exert effects in the gut remain unknown. One animal study has found an increase of *Wickerhamomyces* in the gut of mice with DSS-induced colitis [12]. However, in this study, we found that the *Wickerhamomyces* is positively associated with intestinal inflammation in patients with UC (Figure 6). Several studies have reported that *Penicillium* is increased in the gut of IBD patients [14, 18]; however, some species of *Penicillium* (e.g., the penicillin producer *A. chrysogenum* and a new *Penicillium sp*.) are known to have antibacterial and/or antifungal activities, while other *Penicillium* species produce metabolites that induce inflammatory responses in cells and are harmful to humans [18]. The genera *Sporobolomyces* and *Trametes* were found to be negatively correlated with intestinal inflammation. *Sporobolomyces singularis* was reported to be a producer of prebiotics enzymes [25], revealing the potential beneficial role of *Sporobolomyces* in the gut. Lim [26] showed that *Trametes versicolor* has anti-inflammatory properties in attenuating colitis in murine IBD. Whether other *Trametes* species have similar anti-inflammatory properties in the human gut requires further investigation. Therefore, *Sporobolomyces* and *Trametes* may represent two probiotic fungal genera in the human gut; however, this requires further investigation. It is interesting that although the proportion of *Aspergillus* increased significantly in the gut of UC patients compared with that in the HS, it did not show a positive correlation with the inflammatory cytokines and/or clinical inflammatory index. Moreover, the number of fungi (18S rDNA level) and Shannon index were not significantly correlated with the intestinal inflammatory status.

The intestinal microbiota is thought to be an important factor in the development of IBD. Numerous studies have reported an association between intestinal bacteria and gut inflammation [27]; however, other types of microorganisms (e.g., fungi and viruses) that colonize the human gut have not been thoroughly investigated. The results of this study deepen the present understanding of the role of intestinal mucosal fungi in HS and UC patients. However, this study has some limitations. First, the sample size was limited, and the samples were only collected from the inflamed mucosa and not from non-inflamed mucosa or feces. Thus, we were unable compare the inflamed mucosal-associated fungi with their non-inflamed or fecal counterparts. Second, the mucosal biopsies were only acquired from the descending colon of each patient. This may have prevented us from obtaining all relevant biological information from the gut. Third, some patients in this study were not new-onset patients and had received mesalazine therapy, and enrolled patients had different degrees of disease, which may have affected their gut microbial composition. In this study, owing to the limitation of the sample size, no significant differences were found between UC patients who received or did not received mesalazine, between patients with mild and moderate-to-severe colitis, and between patients with and without new-onset colitis. We believed that a larger sample size should reveal additional differences between these groups in future studies. Nevertheless, this study provides a better understanding of the shift of fungal genera and their potential in promoting intestinal inflammation in patients with UC. As it is believed that mucosa-associated microbiota is more relevant to the pathogenesis of IBD than fecal microbiota, we studied the association between intestinal mucosal fungi and intestinal inflammation in this study and uncovered a potential link between the intestinal fungi and IBD pathogenesis.

In conclusion, this study confirms an alteration of mucosal fungal community in UC patients compared with HS, and puts forward the hypothesis that several specific fungal genera (and/or species) play important roles in intestinal inflammation. Further studies should be performed to evaluate the role of intestinal fungi in IBD pathogenesis more precisely and examine their potential utility as therapeutics targets.

## MATERIALS AND METHODS

### Study population

This study was approved by the Administrative Panel for Medical Research on Human Subjects of the First Affiliated Hospital of Nanjing Medical University, and written informed consent was obtained before study enrollment. In total, 14 UC patients and 15 healthy controls were recruited. All patients were diagnosed with UC according to established clinical, endoscopic, radiologic, and histologic criteria [28]. None of the study participants had taken probiotics, prebiotics, antibiotics, anti-fungal agents, or colon-cleansing regimens for at least 8 weeks prior to enrollment [14]. None of the subjects had a prior history of metabolic disease or gastroenterology surgery. Female subjects who were pregnant or lactating were excluded. UC patients received only mesalazine or no therapy before study enrollment. Control group were constituted by HS that who had normal colonoscopy performance and did not have a history and clinical symptoms of intestinal disorders. Subjects characteristics are listed in Table 1.

### Assessment of ulcerative colitis activity

The endoscopic severity for UC patients was evaluated by the modified Baron's score ranges from 0 to 4 as described previously [29], with 0 = denoting normal mucosa, 1 = granular mucosa with an abnormal vascular pattern, 2 = friable mucosa, 3 = microulceration with spontaneous, 4 = gross ulceration. The disease activity of UC was assessed by the Mayo score (consisting of stool frequency, rectal bleeding, findings from flexible proctosigmoidoscopy, and physician's global assessment of disease) [30]. A higher score representing an increased degree of inflammation (Table 2).

### Sample collection

Three biopsy samples of inflamed descending colon from UC patients and the normal descending colon from HS were collected respectively during endoscopic biopsies. All the mucosal samples were immediately placed in liquid nitrogen and stored at –80°C until further processing [12].

### DNA isolation and library construction

DNA was extracted from one colonic mucosa biopsy using a FastDNA^®^ SPIN Kit for Feces (MP Biomedicals) according to the method described before and quantified on a NanoDrop 1000 spectrophotometer (Termo Scientifc) [12]. The internal transcribed spacer regions 1 and 2 (ITS1-2, representing fungi) genes were amplified for sequence analysis as previously described [12].

### Bioinformatics analysis

The Illumina HiSeq 2000 platform (reconstructed cDNA sequence: 2 × 150 bp) was applied to detect the amplified ITS1-2 DNA sequences in the mucosal specimens. Primer sets were modified with Illumina adapter regions for sequencing on the Illumina GAIIx platform, and reverse primers were modified with an 8-bp Hamming error-correcting barcode to distinguish the different samples. The Ribosomal Database Project (RDP) Classifier was used for taxonomical assignment after the raw sequences were identified by their unique barcodes [12]. Fungal genera with an average relative abundance of ≥ 0.01 in samples were considered to be the major genera. Operational taxonomic units (OTUs) present in ≥ 33.3% of the mucosal specimens from UC patients and/or HS were identified as core OTUs and were subsequently analyzed by partial least-squares discriminant analysis (PLS-DA) and clustered by Simca-P version 12 (Umetrics) into different groups [31]. *Heat maps* were generated in TMEV *Multiple Experiment Viewer* v4.5.1 software based on the core OTUs of fungal communities. Community diversity was measured by the Shannon-Weiner biodiversity index (Shannon index) [32].

### Real-time PCR

To determine the levels of pro-inflammatory cytokines (IL-Iβ, TNF-α, INF-γ, IL-6, IL-17A, and IL-23) in the colonic mucosa by the Real-time PCR method, total RNA was extracted from mucosal biopsy samples using the TRIzol method (Gibco) and converted into cDNA. RPLPO, which has been reported to be stable in the inflammatory gut [33], was used as the internal control. The primers used are listed in Table 3. To measure the fungal concentrations in mucosa, primers for 18S (representing fungi) were used for the PCR experiment [11], and the amounts of 18S rDNA were calculated using standard curves constructed with known concentrations of plasmid DNA (Transgene, Beijing, China) containing the respective sequences to be amplified [12].

**Table 3:** Primers used for the pro-inflammatory cytokines (IL-1β, TNF-α, IFN-γ, IL-6, IL-17A, IL-23) and 18S rDNA level (representing fungi concentration) detecting in mucosal biopsy samples of UC patients and healthy subjects. RPLPO was used as the housekeeping gene. The internal transcribed spacer regions 1 and 2 (ITS1-2) gene was amplified for sequence analysis of the fungal composition

Amplicon	Forward primer	Reverse primer
IL-1β	ATGATGGCTTATTACAGTGGCAA	GTCGGAGATTCGTAGCTGGA
TNF-α	TCTGGCCCAGGCAGTCAGATC	TCAGCTTGAGGGTTTGCTACAA
IFN-γ	TTTTAATGCAGGTCATTCAGATGT	AAGTTTGAAGTAAAAGGAGACAATTTGG
IL-6	CCAGGAGCCCAGCTATGAAC	CCCAGGGAGAAGGCAACTG
IL-17A	TGATTGGAAGAAACAACGATGACT	ATTGTGATTCCTGCCTTCACTATG
IL-23	CCCAAGGACTCAGGGACAAC	TCCTAGCAGCTTCTCATAAAAAATCA
18S rDNA	ATTGGAGGGCAAGTCTGGTG	CCGATCCCTAGTCGGCATAG
RPLPO	GCGACCTGGAAGTCCAACTA	TCTCCAGAGCTGGGTTGTTT
ITS1-2	CTTGGTCATTTAGAGGAAGTAA	GCTGCGTTCTTCATCGATGC

### Statistical analyses

Fungal community composition was analyzed at the phylum, genus, and species levels. The overall differences in the fungal microbiota composition in colonic mucosa biopsies between normal and UC group were compared, and results are expressed as the mean ± standard error of the mean (SEM). Differences between the two groups were analyzed by variance (ANOVA) and Mann-Whitney *U*-tests. Spearman correlation analysis was used to analyze the correlation between intestinal fungal composition and intestinal inflammatory status. Statistical analyses were performed using the Statistical Package for Social Sciences version 19.0 (SPSS Inc., Chicago, IL).

## SUPPLEMENTARY MATERIALS FIGURES AND TABLES




