# Supplementary material for: Alterations in the mucosa-associated fungal microbiota in patients with ulcerative colitis
Source: Oncotarget. 2017 Nov 20;8(64):107577–88. doi: 10.18632/oncotarget.22534 (PMC5746090; doi:10.18632/oncotarget.22534)
Supplement: Supplementary file 1 [file oncotarget-08-107577-s001.pdf]

Alterations in the mucosa-associated fungal microbiota in patients with ulcerative colitis

SUPPLEMENTARY MATERIALS

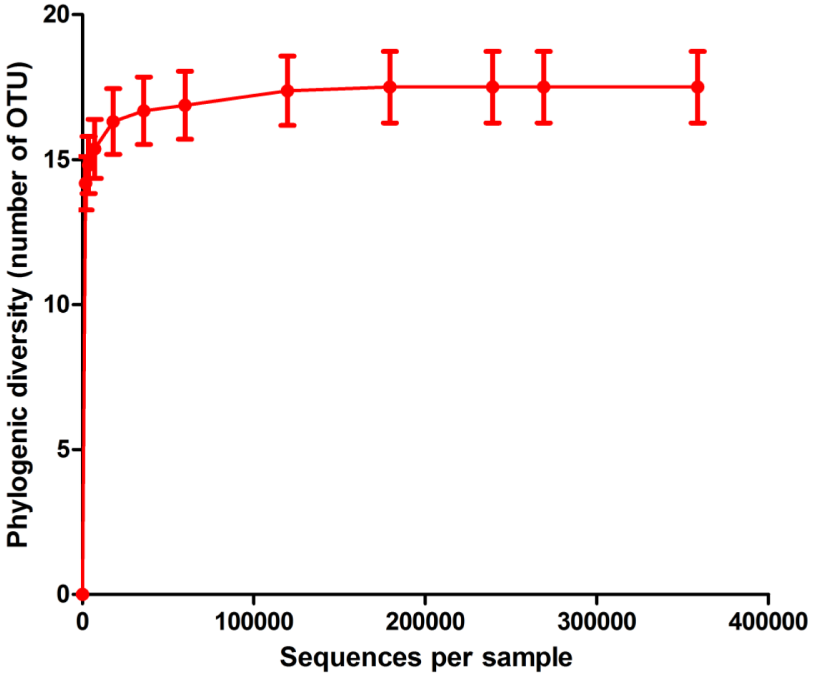

Supplementary Figure 1: Rarefaction analysis of sampling by observed fungal OTU method.

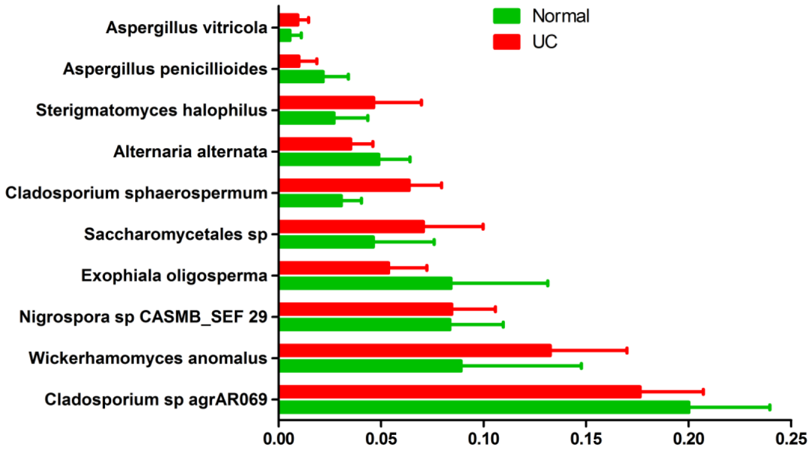

Supplementary Figure 2: Comparisons of 10 main bacterial species (relative abundance ≥ 0.01 on average) in the colonic mucosa of HS and UC patients.

Supplementary Table 1: Nineteen core OTUs of fungal communities inferred from colonic mucosa in HS and UC patients. See Supplementary_Table_1.
